# Efficacy of *Carapa guianensis* oil (Meliaceae) against monogeneans infestations: a potential antiparasitic for *Colossoma macropomum* and its effects in hematology and histopathology of gills

**DOI:** 10.1590/S1984-29612023051

**Published:** 2023-09-01

**Authors:** Dayna Filocreão Malheiros, Marcela Nunes Videira, Abthyllane Amaral Carvalho, Clara Brito Salomão, Irlon Maciel Ferreira, Kirley Marques Canuto, Eliane Tie Oba Yoshioka, Marcos Tavares-Dias

**Affiliations:** 1 Programa de Pós-graduação em Biodeversidade Tropical – PPGBIO, Universidade Federal do Amapá – UNIFAP, Macapá, AP, Brasil; 2 Universidade do Estado do Amapá – UEAP, Macapá, AP, Brasil; 3 Laboratório de Biocatálise e Síntese Orgânica Aplicada, Universidade Federal do Amapá – UNIFAP, Macapá, AP, Brasil; 4 Embrapa Agroindústria Tropical, Fortaleza, CE, Brasil; 5 Embrapa Amapá, Macapá, AP, Brasil

**Keywords:** Aquaculture, phytotherapy, monogenean, treatment, Aquicultura, fitoterapia, monogenético, tratamento

## Abstract

This study evaluated the efficacy of therapeutic baths with *Carapa guianensis* (andiroba) oil against monogeneans of *Colossoma macropomum* (tambaqui), as well as the hematological and histological effects on fish. Among the fatty acids identified in *C. guianensis* oil, oleic acid (53.4%) and palmitic acid (28.7%) were the major compounds, and four limonoids were also identified. Therapeutic baths of 1 hour were performed for five consecutive days, and there was no fish mortality in any of the treatments. Therapeutic baths using 500 mg/L of *C. guianensis* oil had an anthelmintic efficacy of 91.4% against monogeneans. There was increase of total plasma protein and glucose, number of erythrocytes, thrombocytes, leukocytes, lymphocytes and number of monocytes and decrease in mean corpuscular volume. Histological changes such as epithelium detachment, hyperplasia, lamellar fusion and aneurysm were found in the gills of tambaqui from all treatments, including controls with water of culture tank and water of culture tank plus iso-propyl alcohol. Therapeutic baths with 500 mg/L of *C. guianensis* oil showed high efficacy and caused few physiological changes capable of compromising fish gill function. Results indicate that *C. guianensis* oil has an anthelmintic potential for control and treatment of infections by monogeneans in tambaqui.

## Resumo

Avaliou-se a eficácia de banhos terapêuticos com óleo de *Carapa guianensis* (andiroba) contra monogenéticos de *Colossoma macropomum* (tambaqui), bem como os efeitos hematológicos e histológicos. Dentre os ácidos graxos identificados no óleo de C*. guianensis*, ácido oleico (53,4%) e ácido palmítico (28,7%) foram os compostos majoritários, e quatro limonoides também foram identificados. Banhos terapêuticos de 1 hora foram realizados por cinco dias consecutivos, não havendo mortalidade de peixes em nenhum dos tratamentos. Banhos terapêuticos, com 500 mg/L de óleo de *C. guianensis*, apresentaram eficácia de 91,4% contra monogenéticos. Houve aumento dos níveis plasmáticos de proteína total e glicose, número de eritrócitos, trombócitos, leucócitos, linfócitos e número de monócitos e diminuição do volume corpuscular médio. Alterações histológicas, como descolamento do epitélio, hiperplasia, fusão lamelar e aneurisma, foram encontradas nas brânquias de tambaqui de todos os tratamentos, incluindo os controles com água do tanque de cultivo e água do tanque de cultivo + álcool isopropílico. Banhos terapêuticos com 500 mg/L de óleo de *C. guianensis* mostraram alta eficácia e causaram poucas alterações fisiológicas capazes de comprometer a função branquial dos peixes. Esses resultados indicam que o óleo de *C. guianensis* apresenta potencial anti-helmíntico para controle e tratamento de infecções causadas por monogenéticos em tambaqui.

## Introduction

Aquaculture is one of the fastest growing agricultural activities in the world, contributing to more than half of the fish consumed, generating employment and income in many countries (FAO, 2022). In recent years, this growth may be related to the adoption of new techniques that reflect in the increase of production quality and productivity. These productivity gains should support expectations for increased production in the coming decades, through sustainability and increased production areas. However, parasitic diseases continue to be obstacles to the development of industrial fish farming (Soler-Jiménez et al., 2017; Tavares-Dias & Martins, 2017). In particular, diseases caused by monogenetic parasites have resulted in economic losses for fish farming, as intense infestations reduce fish growth, thereby reducing commercial value, in addition to increasing the mortality rate and treatment costs (Gomes et al., 2017; Tavares-Dias & Martins, 2017; Pimentel-Acosta et al., 2019).

Treatment and control of infections caused by monogeneans in fish farms, in general, has been carried out using different chemotherapeutics, whose prolonged and frequent use can pose a risk to the health of fish, the environment and humans, because of the accumulation of chemical residues in the meat of the fish (Malheiros et al., 2016; Hashimoto et al., 2016; Tavares-Dias & Martins, 2017; Malheiros et al., 2020). Thus, current studies have focused on the use of medicinal plants and their derivatives as an alternative for control and treatment of diseases caused by monogeneans in fish population (Boijink et al. 2015, 2016, Hashimoto et al., 2016; Malheiros et al., 2016; Soares et al., 2016, 2017; Barriga et al., 2020; Luz et al., 2021; Malheiros et al., 2022). Oils of medicinal plants are more ecologically friendly, in addition to having several bioactive components such as polysaccharides, organic acids, alkaloids, terpenoids, glycosides, volatile oils, oleoresin, among other compounds (Tavares-Dias, 2018; Barriga et al., 2020; Doan et al., 2020; Luz et al., 2021), which can have anthelminthic effects.

*Carapa guianensis* Aublet) Steudel, 1821 is a plant of the Meliaceae family popularly known in Brazil as andiroba, and widely used in popular medicine, especially in northern Brazil, whose oil, extracted from the seeds, stands out due to its commercial and therapeutic importance due to its medicinal properties (Farias et al., 2007, 2010; Lira-Guedes & Nardi, 2015). Diverse studies suggest that the pharmacological properties of this oleoresin are attributed to the various limonoids present in its composition (Matsui et al., 2014; Henriques & Penido, 2014; Marques et al., 2016; Oliveira et al., 2018; Nascimento et al., 2019). *In vitro* efficacy of *C. guianensis* has been reported against larvae of nematodes *Haemonchus* sp., *Oesophagostomum* sp. and *Trichostrongylus* sp. of goats and sheep (Farias et al., 2010) and *Boophilus microplus* (Farias et al., 2007). *In vitro* antiplasmodial activity of *C. guianensis* oil, due to the presence of limonoides gedunin, was dependent on the exposure time (Marques et al., 2016). However, this oil has not been used in fish species. Thus, this study aimed to investigate the anthelmintic efficacy of therapeutic baths with *C. guianensis* oil against monogeneans from the gills of *Colossoma macropomum* (tambaqui), as well as to evaluate the hematological and histological effects in this host fish.

## Materials and Methods

### Obtention and composition of fatty acids and limonoids from *C. guianensis* oil

*Carapa guianensis* oil was supplied by the Kamukaia III project, from Embrapa Amapá (Brazil) and an aliquot of this oil was submitted to analysis, where the fatty acids were converted into fatty acid methyl esters (FAMEs) following the method of Hartman & Lago (1973). After extraction, the materials were analyzed by Chromatograph equipped with a Flame Ionization Detector (GC2010 Plus, Shimadzu, Kyoto, Japan) and a stationary phase biscianopropyl-polydimethylsiloxane capillary column (SP2560, 100 m × 0.25 mm, df 0.20 Supelco^®^, Bellefonte, PA, USA). The oven temperature of the column was as follows: the initial temperature was maintained at 80 °C, increased to 11°C/min at 180°C and 5°C/min at 220 °C and then held for 23 min. Hydrogen was used as a carrier gas at a flow rate of 1.5 mL/min, the split ratio was 1:30, and the injector and detector temperatures were 220 °C. FAMEs were identified by a comparison of the retention times with those of a previously injected fatty acid standard mix (code CRM47885, Supelco^®^, Bellefont PA, USA), following the same methodology. The contribution of each compound to the mixture was given by the relative area (%) of its respective peak in the chromatogram (FAME, Supelco^®^, Bellefont PA, USA). The contribution of each compound to the mixture was given by the relative area (%) of its respective peak in the chromatogram.

Five grams of *C. guianensis* oil were chromatographed on a silica gel glass column (5 g), eluting with 40 mL of 95:5 CHCl3-hexane (FCH), followed by 25 mL of 95 CHCl3-acetonitrile: 5 (FCA) and finally 25 mL of acetonitrile (FA), to obtain the limonoids. The fractions were rotoevaporated, yielding: 3.73 g; 1.19 g and 69.3 mg, respectively. Then, an aliquot of FCA (108 mg) was dissolved in 400 µL of methanol (MeOH) and subjected to solid phase extraction (1g SPE-C18 cartridge). The sample was eluted with 5 mL of a water-methanol mixture (H2O/MeOH) with 50, 60, 70, 80 and 90% methanol, ending with 100% methanol. The collected fractions were rotoevaporated at 40^o^C and dried with nitrogen gas flow, resulting in 50% FCA-H2O/MeOH (0.3 mg); 60% FCA-H 2 O/MeOH (0.6 mg); 70% FCA-H 2 O/MeOH (1.4 mg); 80% FCA-H 2 O/MeOH (2.2 mg); 90% FCA-H 2 O/MeOH (3.4 mg) and 100% FCA-MeOH (30.6 mg).

The samples obtained of *C. guianensis* oil by SPE were analyzed using an Acquity UPLC^®^ system consisting of a UPLC liquid chromatograph coupled with a quadrupole mass spectrometer and a diode array detector (UPLC-Q-Da, Waters, Milford, MA, USA). Samples were previously filtered through 0.22 μm PTFE membranes (Millipore^®^) with chromatographic runs performed on a Waters BEH C18 column (150 mm x 2.1 mm, 1.7 μm) at 40°C and an injection volume of 5μl. The mobile phase consisted of 0.1% formic acid in water (A) and 0.1% formic acid in acetonitrile (B), with a flow rate of 0.3 mL/min. The elution gradient was as follows: 0 min, 2% (B); 22.0 min, 95% (B); 22.5 min, 100% (B); 25.0 min, 100% (B); 26.0 min, 2% (B); 30 min, 2% (B). The ionization mode was performed by positive ESI with the following parameters: desolvation gas, N2, 600 L/h, extraction cone voltage of 10V, capillary voltage of 1.2 kV.

### Fish and acclimatization

Juveniles of *C. macropomum* (17.7 ± 4.91 cm and 88.3 ± 56.3 g) were acquired from a commercial fish farm in Macapá, in the state of Amapá (Brazil) and transported to the Aquaculture and Fisheries Laboratory of Embrapa Amapá, Macapá, Amapá (Brazil). The fish were kept in a 1000 L tank, with constant aeration and continuous water flow, and fed with rations containing 32% crude protein twice a day, until use in the tests. These fish, naturally parasitized by monogeneans, were used in all trials.

The following water quality parameters were monitored daily: temperature (29.7 ± 0.1 °C), dissolved oxygen (5.5 ± 0.2 mg/L), pH (5.8 ± 0.2), ammonia (0.4 ± 0.2 mg/L), alkalinity (10.0 ± 0.001 mg/L) and hardness (10.0 ± 0 mg/L), with the aid of a multiparameter probe (YSI, USA). The tank was siphoned weekly to remove organic matter accumulated at the bottom.

### Tolerance test of *C. macropomum* to different concentrations of *C. guianensis* oil

Tolerance tests were performed with *C. macropomum* (17.7 ± 4.91 cm and 88.3 ± 56.3 g) to determine the ideal concentration for therapeutic baths with *C. guianensis* oil. Sixty of *C. macropomum* were distributed in 100 L water tanks (80 L volume) with three replicates and five fish in each replicate (15 fish per treatment). The treatments were carried out with different concentrations of *C. guianensis* oil (300, 500, 800 and 1,000 mg/L). Iso-propyl alcohol was used as solvent (1:10 g) for *C. guianensis* oil. The tanks used in the tolerance tests were kept without water renewal. Fish were observed during 4 h of exposure to *C. guianensis* oil, for changes in fish behavior (i.e., opercular movement, caudal beating, erratic swimming, response to mechanical stimuli) and/or mortality.

### Therapeutic baths with *C. guianensis* oil against monogeneans of *C.macropomum*

A total of 117 of *C. macropomum* (17.7 ± 4.91 cm and 88.3 ± 56.3 g), naturally parasitized by monogeneans, were distributed in 100 L water tanks, maintained with a static water system and with constant aeration. The experimental design consisted of three treatments, with three replicates containing 13 fish in each replicate (39 fish per treatment), with two controls (one with tank water only, the other with tank water + iso-propyl alcohol), in addition to the best tolerance test result was 500 mg/L of *C. guianensis* oil. Baths with *C. guianensis* oil were of 1 h for five consecutive days.

After the last therapeutic bath, the fish were euthanized by medullary section and gills of 10 fish from each replicate (30 fish per treatment) were collected and fixed in 5% formalin for quantification and identification of monogeneans (Eiras et al., 2006), and determination of prevalence, mean intensity and mean abundance (Bush et al., 1997). The effectiveness of therapeutic baths was determined using methodology previously described by Zhang et al. (2014).

### Blood parameters of *C. macropomum* exposed to *C. guianensis* oil

After the therapeutic baths, five fish were collected from each of the three replicates (15 fish per treatment) to evaluate the blood parameters of animals exposed to 500 mg/L of *C. guianensis* oil and controls. Blood samples were collected by puncturing the caudal vessel, using syringes containing EDTA (10%). Blood was used for the following determinations: hematocrit (Ht) by the microhematocrit method, total erythrocytes count (RBC) using a Neubauer chamber and hemoglobin concentration ([Hb]) by the cyanomethemoglobin method. Hematimetric indices, mean corpuscular volume (MCV) and mean corpuscular hemoglobin concentration (CHCM) were calculated from the values of Ht, RBC and [Hb]. Blood smears were prepared and panchromatically stained with a combination of May Grünwald-Giemsa-Wright, for the differential count of leukocytes in up to 200 cells of interest, in each smear. Blood smears were also used to determine the number of total leukocytes and thrombocytes (Ranzani-Paiva et al., 2013).

The rest of the blood was centrifuged to obtain blood plasma, for the analysis of glucose levels by the enzymatic-colorimetric method of glucose oxidase and total proteins by the biuret method, using kits (Doles, GO, Brazil) and readings in a UV/Visible spectrophotometer reading.

### Histopathology of the gills *C. macropomum* exposed to *C. guianensis* oil

At the end of the therapeutic baths with *C. guianensis* oil, three fish from each of the three replicates (9 fish per treatment) were euthanized by medullary section and the first right and left gill arches were collected for histopathological analysis. The gills were fixed in Davidson's solution (95% alcohol, acetic acid, formaldehyde and distilled water) for at least 24 h, and then transferred to ethyl alcohol (70%). Subsequently, the gill arches were dehydrated in ascending order of alcohol (70%, 80%, 90%, absolute I, II and III), diaphanized in 100% xylene concentration, impregnated and embedded in paraffin to obtain the blocks. The paraffin blocks were cut in the microtome (Leica DM 1000) with 5 µm thick. After making the slides (in duplicates), they were stained with Hematoxylin and Eosin (HE). The images were captured with a digital camera (Moticam 2300 3.0 M Pixel) attached to a common optical microscope, connected to the computer. The histopathological analyzes performed were semiquantitative, using the mean assessment values (MAV) (Schwaiger et al., 1997) and the histopathological alteration index (HAI) (Poleksic & Mitrovic-Tutundzic, 1994).

### Statistical analysis

All parasitic, blood and histopathological data were previously evaluated to normality and homoscedasticity using Shapiro-Wilk and Bartlett, respectively. As the data did not follow a normal distribution, the Kruskal-Wallis test was used, and the differences between the medians were compared using the Dunn test (Zar, 2010).

## Results

### Composition of *C. guianensis* oil

Among the fatty acids identified in *C. guianensis* oil, oleic acid (53.4%) and palmitic acid (28.75%) were the major compounds (Table 1).

**Table 1:** Fatty acid composition of *Carapa guianensis* oil.

**Fatty acids**	**Relative percentage**
Palmitic (C16:0)	28.75 ± 0.27
Palmitoleic (C16:1)	0.75 ± 0.03
Stearic (C18:0)	7.66 ± 0.06
Oleic (C18:1)	53.4 ± 0.08
Linoleic (C18:2)	8.11 ± 0.01
Arachidonic (C20:4)	1.08 ± 0.09
Others	0.25 ± 0.01
Total saturated	36.41
Total monounsaturated	54.15
Total polyunsaturated	9.19

Analysis by UPLC-QDA, in positive ionization mode, allowed the identification of four limonoids, in addition to two unidentified derivatives (Table 2). The estimated content of total limonoids was 8.8 mg limonoids/g of oil.

**Table 2:** Chemical characterization of SPE-C18 fractions from *Carapa guianensis* oil based on analysis in a UPLC liquid chromatograph coupled with a quadrupole mass spectrometer and diode array detector, in positive ionization mode.

**Peak**	**Rt (min)**	**[M+H]^+^**	**Molecular formula**	**Compound**	**FCA-50% MEOH**	**FCA-60% MEOH**	**FCA 70% MEOH**	**FCA-80% MEOH**	**FCA-90% MEOH**	**FCA-10% MEOH**
1	14.50	299	-	Unknown	-	-	X	X	-	-
2	15.14	439	C_26_H_30_O_6_	Deacetyloxogedunin	-	-	X	X	-	-
3	15.76	471	C_27_H_34_O_7_	Methyl angolensate	-	-	X	X	-	-
4	16.33	541	C_30_H_36_O_9_	6α-Acetoxy-gedunin	-	-	X	X	-	-
5	16.47	483	C_28_H_34_O_7_	Gedunin	-	-	-	X	-	-
6	17.47	467	-	Limonoid derivative	-	-	-	X	X	-
7	21.79	255	-	Unknown	-	-	-	X	X	-

### Tolerance of *C. macropomum* to different concentrations of *C. guianensis* oil

During the tolerance tests, the fish reacted with agitation to the first contact with all concentrations of *C. guianensis* oil tested. At concentrations of 300 and 500 mg/L, some fish were grouped together at the bottom of the tanks, while others continued to swim actively. At the highest concentrations (800 and 1,000 mg/L) the fish fell to the bottom of the tanks and showed slow breathing and mouth opening. There was no mortality at any of the concentrations tested and at the end of 4 h of exposure all fish were already swimming actively.

### Therapeutic baths with *C. guianensis* oil against monogeneans of *C. macropomum*

There was no *C. macropomum* mortality during or after the five therapeutic baths, on consecutive days. The prevalence, mean intensity and mean abundance of monogeneans (*Anacanthorus spathulatus*, *Notozothecium janauachensis* and *Mymarothecium boegeri*) were decrease (p<0.05) at the gills of the fish exposed to 500 mg/L of *C. guianensis* oil. There were no differences (p>0.05) in prevalence and mean abundance between the two control groups: water from the culture tank, and water from the culture tank + iso-propyl alcohol (Table 3).

**Table 3:** Parasitological index of *Colossoma macropomum* exposure to *Carapa guianensis* oil and efficacy of the therapeutic baths.

Treatments	Prevalence (%)	Mean abundance	Mean Intensity	Efficacy (%)
Water	100	121.6 ± 173.2^a^	121.6 ± 173.2^a^	-
Iso-propyl alcohol	100	81.2 ± 79.8^a^	81.2 ± 79.8^a^	33.2
500 mg L	53.3	6.0 ± 10.2^b^	11.3 ± 11.4^b^	91.4

Data are expressed as mean ± standard deviation. Different letters in the same column indicate significant difference by Dunn test (p<0.05).

Therapeutic baths with 500 mg/L of *C. guianensis* oil showed 91.4% anthelmintic efficacy against monogeneans of *C. macropomum* gills (Table 3).

### Blood parameters of *C. macropomum* exposed to *C. guianensis* oil

In fish exposed to 500 mg/L of *C. guianensis* oil, plasma levels of glucose and total protein, number of erythrocytes, thrombocytes, leukocytes, lymphocytes, monocytes and eosinophils increased (p<0.05) when compared to the control with water of the culture tank, while the VCM decreased. In fish exposed to tank water + iso-propyl alcohol, there was an increase (p<0.05) in plasma glucose levels, number of erythrocytes, thrombocytes, leukocytes, lymphocytes, monocytes and neutrophils when compared to the control with tank water culture. And comparing the fish exposed to tank water + iso-propyl alcohol to those exposed to andiroba oil, the plasma levels of glucose and total protein, number of erythrocytes, thrombocytes, leukocytes, lymphocytes, monocytes and eosinophils were similar. The other parameters evaluated showed no changes among treatments (Table 4).

**Table 4:** Blood parameters of *Colossoma macropomum* exposed to *Carapa guianensis* oil.

**Parameters**	**Water**	**Iso-propyl alcohol**	**500 mg L**
Glucose (mg/dL)	117.3 ± 82.03^a^	146.0 ± 22.74^b^	143.1 ± 28.77^b^
Total protein (g/dL)	2.8 ± 0.18^a^	2.9 ± 0.21^a,b^	3.2± 0.45^b^
Erythrocytes (x10^6^/μL)	1.60 ± 0.20^a^	3.68 ± 0.66^b^	3.45 ± 0.36^b^
Hemoglobin (g/dL)	7.7 ± 0.77^a^	8.3 ± 0.99^a^	8.0 ± 0.90^a^
Hematocrit (%)	26.6 ± 2.6^a^	28.1 ± 2.0^a^	28.5 ± 1.6^a^
MVC (fL)	168.0 ± 15.61^a^	78.3 ± 12.30^b^	83.1 ± 8.25^b^
MCHC (g/dL)	29.1 ± 2.76^a^	29.5 ± 2.86^a^	27.9 ± 1.97^a^
Thrombocytes (μL)	47,828 ± 11,337^a^	98,633 ± 25,475^b^	113,636 ± 23,860^b^
Leukocytes (μL)	34,154 ± 10,087^a^	73,173 ± 16,636^b^	65,183 ± 17,062^b^
Lymphocytes (μL)	19,582 ± 7,513^a^	43,783 ± 9,366^b^	38,177 ± 11,756^b^
Monocytes (μL)	4,028 ± 1,520^a^	10,302 ± 4,033^b^	9,748 ± 4,073^b^
Neutrophils (μL)	9,025 ± 4039^a^	16,701 ± 8,000^b^	13,331 ± 5,000^a,b^
Eosinophils (μL)	1,250 ± 959^a^	2,301 ± 1,562^a,b^	3,773 ± 2,308^b^
PAS-GL (μL)	281 ± 421^a^	85 ± 228^a^	154 ± 386^a^

Data are expressed as mean ± standard deviation. Different letters in the same line indicate significant difference by Dunn test (p< 0.05). MCV: mean corpuscular volume; MCHC: mean corpuscular hemoglobin concentration.

### Histopathology of the gills of *C. macropomum* exposed to *C. guianensis* oil

After therapeutic baths with 500 mg/L of *C. guianensis* oil, the histopathological analyzes of the gills showed significant differences (p<0.05) regarding HAI, between the treatment with this oil and control with tank water of culture, but there were not significant changes (p >0.05) regarding the MAV (Table 5).

**Table 5:** Values of histopathological alteration index (HAI) and mean assessment values (MAV) for gills of *Colossoma macropomum* exposed to the oil the *C. guianensis*.

Treatments	N	MAV	HAI	Severity of the lesions according to the HAI
Water	5	12.4 ± 2.9^a^	15.4 ± 4.3^a^	Mild to moderate of the organ damage
Iso-propyl alcohol	5	11.4 ± 1.5^a^	13.0 ± 7.1^a,b^	Mild to moderate of the organ damage
500 mg/L	5	8.8 ± 1.8^a^	8.6 ± 5.1^b^	Normal function of the organ

Data are expressed as mean ± standard deviation. Different letters in the same line indicate significant difference by Dunn test (p<0.05). MCV: mean corpuscular volume; MCHC: mean corpuscular hemoglobin concentration.

Histopathological analyzes revealed slight changes in the gills of fish exposed to 500 mg/L of *C. guianensis* oil, as well as moderate and slight changes in fish from the control groups (culture tank water and tank water + iso-propyl alcohol). However, these changes did not affect the functioning of the gills, according to HAI index values (Table 5).

**Figure 1:**
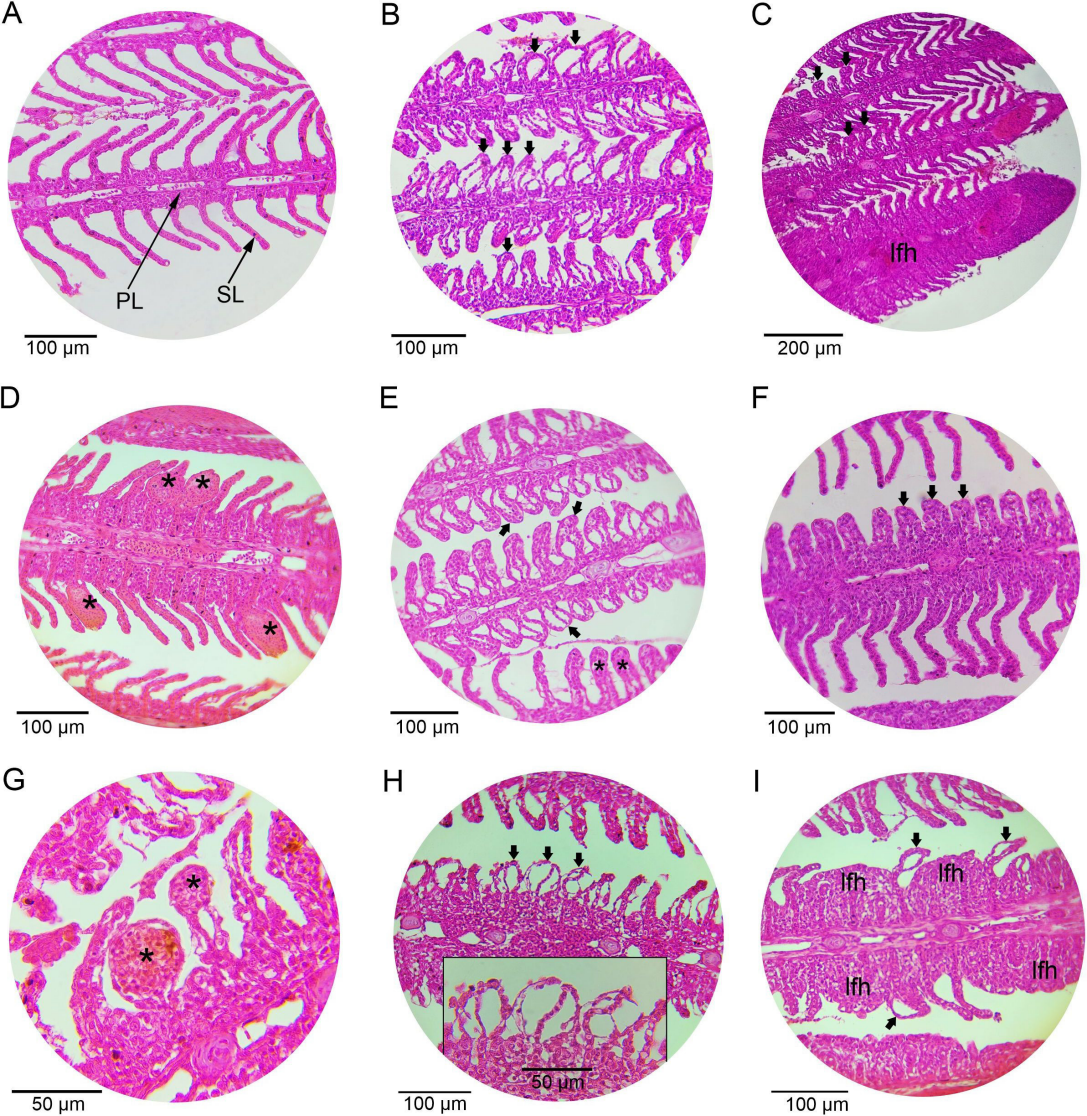
Histopathology of gills from *Colossoma macropomum*. **A-C.** Gills of fish exposed to 500 mg/L from *Carapa guianensis*. **A.** Gills with primary (PL) and secondary (SL) lamellae. **B.** Branchial filaments showing a detachment-type lesion of the lamellar epithelium (arrow). **C.** Branchial filament with lamellar hyperplasia (arrow) and hyperplasia with lamellar fusion (lfh). **D-F.** Gills of fish exposed to culture tank water + isopropyl alcohol. **D.** Branchial filament showing aneurysm-type lesions (asterisk). **E.** Branchial filaments showing lamellar epithelium detachment type lesion (arrow) and lamellar hyperplasia (asterisk). **F.** Branchial filament with secondary filament hyperplasia (arrow). **G-H.** Fish gills exposed to culture tank water (control). **G**. Branchial filament of fish showing aneurysm-type lesions (asterisk). **H.** Branchial filament showing lesions of the lamellar epithelium detachment type (arrow). **I.** Branchial filament showing lesions of the lamellar epithelium detachment type (arrow) and hyperplasia with lamellar fusion (l fh). Staining with hematoxylin and eosin (HE).

The main histopathological changes found in the gills of fish in the control groups were epithelium detachment, hyperplasia, lamellar fusion and aneurysm (Figure 1).

## Discussion

Plant-derived oils are natural products extracted from seeds, bark, roots, leaves, resins or fruits. They are rich in several bioactive compounds, mainly triglycerides (95-98%) (Nascimento et al., 2019; Lüdtke et al., 2021). Regarding the fatty acids of the oil extracted from the seeds of *C. guianensis* oil, in the present study, it was mainly composed of oleic acid (53.4%) and palmitic acid (28.7%). Similar findings were also reported in other studies with oil extracted from the seeds of *C. guianensis* (Matsui et al., 2014; Oliveira et al., 2018; Nascimento et al., 2019; Lüdtke et al., 2021; Sousa et al., 2021). Sousa et al. (2021) cited that the topical use of *C. guianensis* oil was effective in tissue formation, epithelialization, angiogenesis, and collagen deposition in skin lesions. As for limonoid compounds, we identified deacetyloxogedunin, methyl angolensate, 6α-acetoxy-gedunin and gedunin in *C. guianensis* oil. These limonoids were also described in several other studies with *C. guianensis* oil (Miranda Júnior et al., 2012; Henriques & Penido, 2014; Marques et al., 2016; Oliveira et al., 2018; Nascimento et al., 2019). Limonoids are secondary metabolites considered chemical markers for species of the Meliaceae family (Henriques & Penido, 2014; Nascimento et al., 2019), to which *C. guianensis* belongs. The Meliaceae family is rich in limonoids, highly oxygenated tetranortriterpenoid compounds that are reported to possess a wide range of biological activities, such as insecticidal, antifeedant and growth-regulator on insects, antibacterial, antifungal, antimalarial, antiparasitic and antiviral (Miranda Júnior et al. 2012; Henriques & Penido, 2014; Marques et al., 2016; Oliveira et al., 2018; Nascimento et al., 2019).

The toxicity of a therapeutic substance is its property of potentially establishing a pathological state when introduced to an organism or interacting with it. In fish, this can be verified through toxicological evaluation, by obtaining data such as concentration, clinical signs and induced adverse effects (Tavares-Dias, 2018). This first study using *C. guianensis* fixed oil in fish showed that there was no *C. macropomum* mortality at any concentration (300-1,000 mg/L), and at the end of 4 h of exposure all animals were actively swimming. Similar results on the toxicity of *C. guianensis* were also found by Costa-Silva et al. (2008), as they did not observe mortality and signs of toxicity in *Rattus norvegicus* during acute (0.625, 1.25, 2.5 and 5.0 g/kg/day) and subacute (0.375, 0.75 and 1.5 g/kg/day) toxicity tests using this oil for 30 days by via oral. The acute toxicity trial indicated that *C. guianensis* oil of a single dose of 2.0 g/kg orally applied did not produce any sign of toxic effect or death in mice during 14 days (Miranda Júnior et al., 2012). Therefore, high concentrations of *C. guianensis* oil are well tolerated by *C. macropomum*.

Therapeutic baths with 500 mg/L of *C. guianensis* oil, for 1 h per day, for five days, showed 91.4% anthelmintic efficacy against monogeneans from *C. macropomum* gills. Iso-propyl alcohol used as solvent also showed efficacy (33.2%), as has been discussed by Tavares-Dias (2018). Similar efficacy was also reported for *C. macropomum* exposed to *Ocimum gratissimum* essential oil (Boijink et al., 2016), *Alpinia zerumbet* essential oil (Luz et al., 2021) and *Lippia grata* essential oil (Barriga et al., 2020). *In vitro* efficacy of *C. guianensis* oil against larvae of mematodes *Haemonchus* sp., *Oesophagostomum* sp. and *Trichostrongylus* sp. (Farias et al., 2010) and *Boophilus microplus* (Farias et al., 2007) has been reported. Such antiparasitic activities observed with *C. guianensis* oil can be attributed to limonoid-rich fractions (Miranda Júnior et al., 2012; Marques et al., 2016).

Although plant-derived oils (essential, fixed or resin) are recommended for use in the control and treatment of parasitic infections (Tavares-Dias, 2018; Boijink et al., 2016; Barriga et al., 2020; Luz et al., 2021; Malheiros et al., 2022), some species can have adverse effects on the exposed fish (Valladão et al., 2015; Tavares-Dias, 2018; Gonzales et al., 2020). After therapeutic baths with 500 mg/L of *C. guianensis* oil, there was an increase in plasma levels of glucose and total protein, number of erythrocytes, thrombocytes, leukocytes, lymphocytes, monocytes and eosinophils, and a decrease in MCV. Malheiros et al. (2022) also reported increase in levels of plasma glucose and total protein, and number of neutrophils in *C. macropomum* exposed to 100 mg/L of *Copaiba reticulata* oleoresin, for 1 h, or to 250 mg/L of nanoemulsion with this oleoresin, for 2 h. Increases in plasma glucose levels in *C. macropomum* exposed to 60 mg/L of *Cymbopogon citratus* essential oil have been reported (Gonzales et al., 2020). However, in *C. macropomum* exposed to baths with 300 mg/L of *A. zerumbet* essential oil, there was a decrease in the levels of plasma glucose and total protein, and total number of leukocytes, monocytes and neutrophils (Luz et al., 2021). Hyperglycemia is indicative that these baths with oils caused stress in the fish (Urbinati et al., 2020). Leukocytosis in fish exposed to 500 mg/L of *C. guianensis* oil may be related to irritative action and/or damage to the gill epithelium caused by this fixed oil, since leukocytes are responsible for defense (Ranzani- Paiva et al., 2013; Urbinati et al. 2020), especially in cases of infections and/or exposure to irritant substances.

After therapeutic baths of 500 mg/L of *C. guianensis* oil, alterations such as epithelial detachment, lamellar fusion, hyperplasia and aneurysm of all treatments were observed in the gills of *C. macropomum*. Changes such as detachment of the epithelium and hyperplasia imply an adaptive strategy of the fish to increase the distance of the water-blood diffusion process, thus decreasing the absorption of the stressor agent (Winkaler et al., 2007, Shiogiri et al., 2012). In fish exposed to *C. guianensis* oil, the histopathological changes were mild and did not affect the functioning of the gills, while in the control groups (water from the culture tank and water from the culture tank + iso-propyl alcohol) were mild to moderate. Similar results were also reported in studies with *C. macropomum* submitted to therapeutic baths with different essential oils (Malheiros et al., 2016, 2022; Barriga et al. 2020; Gonzales et al., 2020; Luz et al., 2021), and *C. reticulata* oleoresin and nanoemulsion with this oleoresin (Malheiros et al., 2022). However, in *C. macropomum* submitted to a single therapeutic bath with 100 or 150 mg/L of *Lippia alba* essential oil (Soares et al., 2016) and 20 or 40 mg/L with *Lippia origanoides* essential oil (Soares et al., 2017), there were severe and irreversible damages to the gills.

In conclusion, *C. guianensis* oil showed low toxicity to *C. macropomum* and good anthelmintic efficacy against monogeneans from the gills of this host. Under the conditions tested, this oil showed to be safe for *C. macropomum* and well tolerated by fish, in addition the histological findings, suggest a healing action on the gill filaments, which should be investigated. Therefore, the use of 500 mg/L of *C. guianensis* oil can be recommended for controlling and treating infections by monogeneans in *C. macropomum* in five consecutives therapeutic baths.
